# Fast digital zooming system using directionally adaptive image interpolation and restoration

**DOI:** 10.1186/2193-1801-3-713

**Published:** 2014-12-06

**Authors:** Wonseok Kang, Jaehwan Jeon, Soohwan Yu, Joonki Paik

**Affiliations:** Department of Image, Chung-Ang University, Seoul, Korea

**Keywords:** Image interpolation, Image restoration, Edge orientation, Digital zooming

## Abstract

This paper presents a fast digital zooming system for mobile consumer cameras using directionally adaptive image interpolation and restoration methods. The proposed interpolation algorithm performs edge refinement along the initially estimated edge orientation using directionally steerable filters. Either the directionally weighted linear or adaptive cubic-spline interpolation filter is then selectively used according to the refined edge orientation for removing jagged artifacts in the slanted edge region. A novel image restoration algorithm is also presented for removing blurring artifacts caused by the linear or cubic-spline interpolation using the directionally adaptive truncated constrained least squares (TCLS) filter. Both proposed steerable filter-based interpolation and the TCLS-based restoration filters have a finite impulse response (FIR) structure for real time processing in an image signal processing (ISP) chain. Experimental results show that the proposed digital zooming system provides high-quality magnified images with FIR filter-based fast computational structure.

## Introduction

A digital zooming system can increase spatial resolution without a high density image sensor or a high cost optical zoom lens. Recently, most digital cameras adopt a digital zooming system, which is commonly implemented by convolving an up-sampled version of the low-resolution (LR) image with a small kernel using proper weighting coefficients. Popular convolution-based up-sampling methods include linear and cubic-spline interpolation (Wick et al. 2004). Linear interpolation simply averages four neighboring pixels with weights that are inversely proportional to the distance from the target pixel. On the other hand, cubic-spline interpolation determines the pixel intensity value using the weighted average of sixteen neighboring pixels with weights determined by the two dimensional (2D) cubic function. However, neither linear nor cubic-spline interpolation can avoid jagged artifacts in the slanted edge region and blurring artifacts due to the nature of the rectangular-shaped interpolation kernel (Papker et al. 1983; Unser et al. 1991).

To solve this problem, advanced interpolation algorithms have been proposed. Li et al. estimated local covariance coefficients from an LR image and used the estimated coefficients to adapt the interpolation effect based on the geometric duality between the pair of LR and High-resolution (HR) image covariance (Li et al. 2001). Zhang et al. proposed the edge-guided nonlinear interpolation using directional filtering and data fusion (Zhang et al. 2006). Giachetti et al. proposed an up-scaling algorithm based on two-step grid filling and iterative correction of the interpolated pixels by minimizing an objective function depending on the second-order directional derivatives of the image intensity (Giachetti et al. 2011). Zhou et al. proposed the improved cubic-spline interpolation algorithm based on the estimation of the strong edge for a missing pixel location (Zhou et al. 2012). These advanced interpolation algorithms are, however, unsuitable for a fast digital zooming system which has limiter computational power and memory space.

The proposed digital zooming system consists of directionally adaptive image interpolation and restoration. After estimating the edge orientation using steerable filters with edge refinement (Kang et al. 2013a, [b]), the input LR image is adaptively interpolated along the estimated edge orientation using the directionally weighted linear or adaptive cubic-spline interpolation function. The blurring artifacts caused by the interpolation process are then restored using the proposed directionally adaptive truncated constrained least-squares (TCLS) filter (Kim et al. 2009). Both proposed interpolation and restoration filters have a finite impulse response (FIR) structure that is suitable for real-time digital zooming in an image signal processing (ISP) chain.

**Figure 1:**
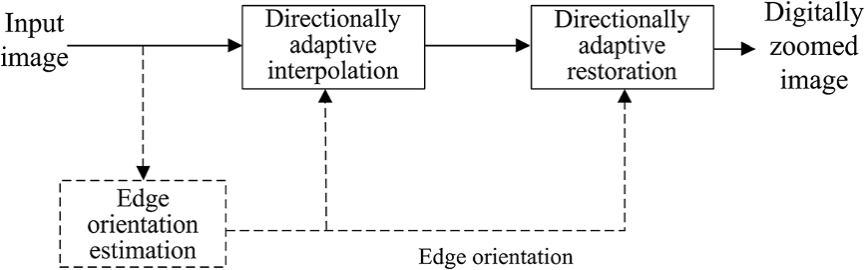
**The block diagram of the proposed digital zooming system.**

The subjective observations show that the proposed method can provide high-quality interpolated images without jagging and blurring artifacts with a high magnification ratio. For objective comparison, the proposed method provides higher peak-to-peak signal-to-noise ratio (PSNR) and structural similarity (SSIM) (Wang et al. 2004) values with lower computation time than existing advanced interpolation algorithms. The block diagram of the proposed digital zooming system is shown in Figure 1.

## The directionally steerable and truncated constrained least-squares (TCLS) filters

The main advantage of the proposed digital zooming system is implemented simply based on the FIR structure for real-time processing in many digital imaging systems. In this section, our proposed four-direction steerable filters and directionally adaptive TCLS restoration filters are described.

### Four-direction steerable filters

In order to determine the edge orientation of an input image, directional steerable filters are used (Freeman et al. 1991). A steerable filter in an arbitrary direction is synthesized using a linear combination of a pair of orthogonal basis filters, such as the 2D circularly symmetric Gaussian function defined in the Cartesian coordinate as
1

where scaling and normalization constants have been set to unity for notational simplicity.

Let *G*^*θ*^ be the 1D derivative of *G*(*x*, *y*) in the direction with angle *θ*. For example, the first-order derivative with *θ* = 0 is expressed as
2

and the same function rotated by 90° is expressed as
3

It is evident that a 1D Gaussian function in an arbitrary orientation *θ* can be synthesized by taking linear combination of  and  as
4

The cos *θ* and sin *θ* terms are used to express an arbitrary direction. For reducing the computational load of edge orientation, four 5 × 5 steerable filters are used with standard deviation *σ* = 1.0. The proposed steerable filter coefficients are generated as shown in Table 1.

**Table 1:** **Directionally steerable filter coefficients**

Angle ***θ***	5 × 5 steerable filter coefficients (***σ*** = 1.0)					
		Horizontal				
	Vertical	-0.0084	-0.0377	-0.0621	-0.0377	-0.0084
		-0.0188	-0.0844	-0.1392	-0.0844	-0.0188
		0.0000	0.0000	0.0000	0.0000	0.0000
		0.0188	0.0844	0.1392	0.0844	0.0188
		0.0084	0.0377	0.0621	0.0377	0.0084
		Horizontal				
	Vertical	-0.0114	-0.0384	-0.0420	-0.0127	0.0000
		-0.0384	-0.1142	-0.0942	0.0000	0.0127
		-0.0420	-0.0942	0.0000	0.0942	0.0420
		-0.0127	0.0000	0.0942	0.1142	0.0382
		0.0000	0.0127	0.0420	0.0382	0.0144
		Horizontal				
	Vertical	0.0084	0.0188	0.0000	-0.0188	-0.0084
		0.0377	0.0844	0.0000	-0.0844	-0.0377
		0.0621	0.1392	0.0000	-0.1392	-0.0621
		0.0377	0.0844	0.0000	-0.0844	-0.0377
		0.0084	0.0188	0.0000	-0.0188	-0.0084
		Horizontal				
	Vertical	0.0000	-0.0127	-0.0420	-0.0382	-0.0144
		0.0127	0.0000	-0.0942	-0.1142	-0.0382
		0.0420	0.0942	0.0000	-0.0942	-0.0420
		0.0382	0.1142	0.0942	0.0000	-0.0127
		0.0114	0.0382	0.0420	0.0127	0.0000

### Directionally adaptive TCLS restoration filter

In order to remove the blurring artifacts caused by the interpolation process, an image restoration filter is needed. Kim et al. proposed the original version of the TCLS restoration filter for removing spatially adaptive image degradation followed by a spatially adaptive noise smoothing filter (Kim et al. 2009). This sub-section presents a directionally adaptive version of the TCLS restoration filter that removes the blurring artifacts caused by the interpolation process.

The input LR image can be considered as a low-pass filtered and sub-sampled version of the original HR image as
5

where *g*(*x*, *y*) and *η*(*x*, *y*) respectively represent 2D arrays of LR image and additive white Gaussian noise. *f*(*p*, *q*) represents the HR image, and *h*(*p*, *q*) the space-variant point spread function (PSF), which plays a role in anti-aliasing filter for the subsequent subsampling operation denoted as *T*[·].

Let *H*(*u*, *v*) be the frequency response of the PSF *h*(*p*, *q*), then the frequency response of the constrained least-squares (CLS) restoration filter is given as (Katsaggelos, 1989)
6

where *C*(*u*, *v*) represents a frequency response of the high-pass filter, and *λ* the regularization parameter the controls the relative amount of data fidelity and the smoothing constraint.

For the CLS filter to become spatially adaptive, five different smoothness according to the edge orientation, *C*^*θ*^(*u*, *v*), for *θ* ∈ {0°, 45°, 90°, 135°, Flat}, are generated using the four directional high-pass filter in the spatial domain as (Kang et al. 2013a, [b])
789

and
10

For minimizing noise amplification in the flat region, the following constraint is used.
11

The frequency response of the modified CLS filter is given as
12

where *C*^*θ*^(*u*, *v*), for *θ* ∈ {0°, 45°, 90°, 135°, Flat}, plays a role of directionally adaptive smoothness constraints, and *λ* = 0.2 is experimentally used. The spatial-domain counterpart of  is its inverse DFT expressed as
13

where *F*^- 1^[·] represents the inverse DFT operation. For reducing the computational load of restoration processing,  is truncated an *m* × *m* FIR filter. Table 2 shows five truncated constrained least-squares (TCLS) filter when *m* = 5.

**Table 2:** **Directionally adaptive TCLS filter coefficients**

Angle ***θ***		5 × 5 TCLS filter coefficients (***λ*** = 0.2)				
		Horizontal				
	Vertical	0.0001	-0.0013	-0.0084	-0.0013	0.0001
		-0.0015	-0.0130	0.0636	-0.0130	-0.0015
		-0.0073	0.0826	0.8018	0.0826	-0.0073
		-0.0015	-0.0130	0.0636	-0.0130	-0.0015
		0.0001	-0.0013	-0.0084	-0.0013	0.0001
		Horizontal				
	Vertical	0.0002	-0.0014	-0.0095	-0.0010	0.0002
		-0.0014	-0.0057	0.0686	-0.0130	-0.0010
		-0.0095	0.0686	0.8099	0.0686	-0.0095
		-0.0010	-0.0130	0.0686	-0.0057	-0.0014
		0.0002	-0.0010	-0.0095	-0.0014	0.0002
		Horizontal				
	Vertical	0.0001	-0.0015	-0.0073	-0.0015	0.0001
		-0.0013	-0.0130	0.0826	-0.0130	-0.0013
		-0.0084	0.0636	0.8018	0.0636	-0.0084
		-0.0013	-0.0130	0.0826	-0.0130	-0.0013
		0.0001	-0.0015	-0.0073	-0.0015	0.0001
		Horizontal				
	Vertical	0.0002	-0.0014	-0.0095	-0.0010	0.0002
		-0.0014	-0.0057	0.0686	-0.0130	-0.0010
		-0.0095	0.0686	0.8099	0.0686	-0.0095
		-0.0010	-0.0130	0.0686	-0.0057	-0.0014
		0.0002	-0.0010	-0.0095	-0.0014	0.0002
		Horizontal				
	Vertical	0.0002	-0.0012	-0.0088	-0.0012	0.0002
		-0.0012	-0.0109	0.0701	-0.0109	-0.0012
		-0.0088	0.0701	0.8077	0.0701	-0.0088
		-0.0012	-0.0109	0.0701	-0.0109	-0.0012
		0.0002	-0.0012	-0.0088	-0.0012	0.0002

## Combined directionally adaptive image interpolation and restoration

**Figure 2:**
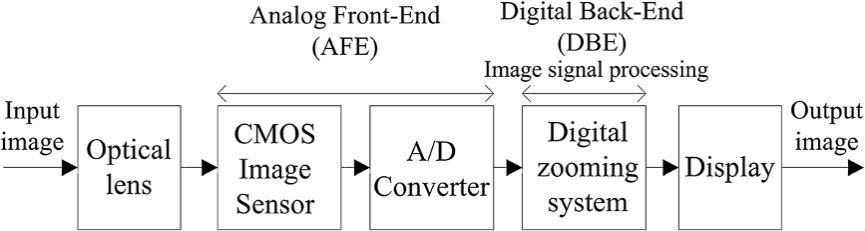
**The image signal processing chain of a digital camera with the proposed digital zooming system in the digital back-end module.**

A typical digital imaging system consists of four functional modules: (i) a set of optical lenses, (ii) the analog front-end (AFE) module including a color filter array (CFA), a complementary metal-oxide-semiconductor image sensor (CIS), and an analog-to-digital converter (ADC), (iii) the digital back-end (DBE) module including various image signal processing subsystems, and (iv) the display devices as shown in Figure 2. The proposed digital zooming system belongs to the DBE module in the ISP chain.

**Figure 3:**
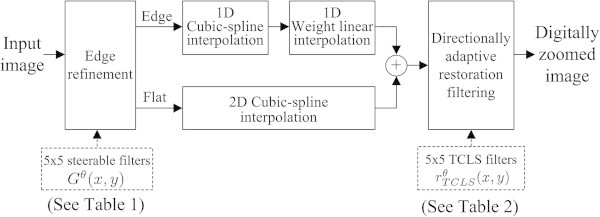
**The block diagram of the proposed digital zooming system combining edge refinement, interpolation, and restoration methods.**

The proposed digital zooming subsystem consists of: (i) estimation of the edge orientation followed by edge refinement, (ii) selective interpolation using either cubic-spline or directionally weighted one-dimensional (1D) linear interpolation along the estimated edge orientation, and (iii) restoration filtering as shown in Figure 3.

### Edge orientation estimation and refinement

In order to determine the edge orientation, the input image is convolved with four 5 × 5 FIR steerable filters given in Table 1 as
14

where *f*_*L*_(*m*, *n*) represents a 5 × 5 local block of the input image centered at (*x*, *y*) and *G*^*θ*^(*x*, *y*) the 5 × 5 FIR steerable filters rotated by angle *θ* ∈ {0°, 45°, 90°, 135°}.

The initial edge orientation is determined by minimizing the mean of *d*^*θ*^(*x*, *y*) as (Kang et al. 2013a, [b])
15

and
16

If the mean value *D*^*θ*^(*x*, *y*) is less than a pre-specified threshold, the corresponding pixel is considered to be in the non-slanted edge region. In this work, the threshold value of 0.075 was used for the empirically optimum sensitivity of steerable filters. Given an initial edge orientation *θ*_*I*_(*x*, *y*), the refined edge orientation is selected among eighteen directions. The proposed edge refinement algorithm is summarized as.


**Figure 4:**
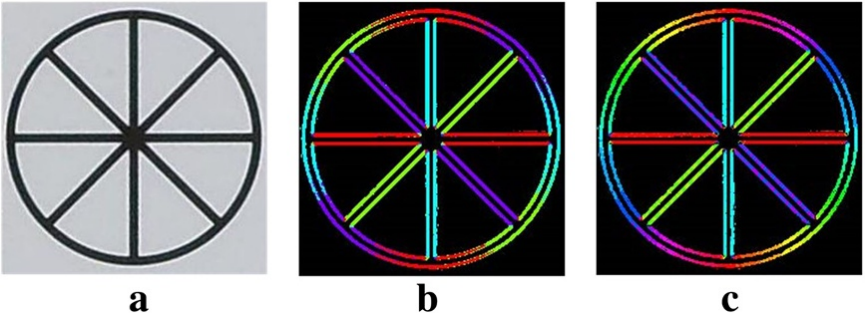
**Results of edge orientation estimation. a** the input image. **b** color coded edge orientation using four directionally steerable filters. **c** color coded edge orientation refined by the proposed method.

The refined edge orientation *θ*^∗^ is finally quantized with the interval of 10°. Figure 4 shows the results of edge orientation estimation using four directionally steerable filters followed by edge refinement. As shown in Figure 4c, the proposed method provides more accurate and continuous edge orientation, which make the result of the proposed directionally adaptive interpolation looks more natural.

### Directionally adaptive image interpolation

**Figure 5:**
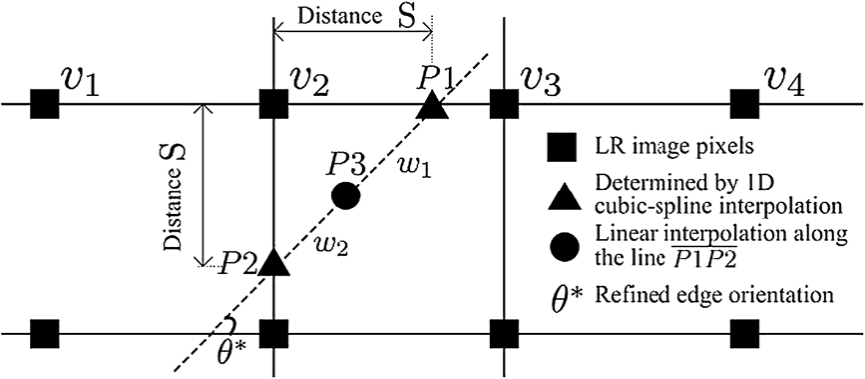
**The proposed directionally adaptive interpolation algorithm.**

In order to interpolate the slanted edge region without jagging artifacts, the proposed method computes the line on the point P3 with refined edge orientation *θ*^∗^ as shown in Figure 5.

The intensity value of P1 is first determined by the proposed adaptive cubic-spline interpolation using the activity-map (Efstratiadis et al. 1990) at *v*_2_ and four pixels on the same horizontal line as
17

where S represents the distance from interpolation point P1, and *f*(·) represents a cubic-spline weight function defined as
18

where *a* represents a cubic-spline weight function parameter.

In this work, the initial parameter value of *a* = - 1 was used. For the spatially adaptive interpolation without blurring artifacts, the cubic-spline weight function parameter is changed according to the strength of the edge using the activity-map (Efstratiadis et al. 1990) as
19

where the tuning parameter *σ* is chosen so that *α*_*MAP*_(*x*, *y*) distributes as uniformly as possible in [0,1], and Var(*x*, *y*) is the local variance of a pixel located at (*x*, *y*). In this work, the tuning parameter of σ = 250 was used*.*

Thus, the adaptive cubic-spline weight function parameter is determined as
20

where *α*_*MAP*_(*x*, *y*) represents the activity value at *v*_2_.

The intensity value of P2 is also determined in the same manner in the vertical direction. Given P1 and P2, the intensity value of P3 is determined by the weighted linear interpolation along the edge line  as (Kang et al. 2013a, [b])
21

where *w*1 represents the distance between P1 and P3, and *w*2 the distance between P2 and P3.

For reducing the computational load of the interpolation process, a simple cubic-spline interpolation is used with *a* = - 0.5, if a pixel is not on the salted edge region. By using the directionally optimized interpolation, the proposed method can significantly reduce jagging artifacts in the slanted edge region.

### Directionally adaptive image restoration

The proposed TCLS restoration filters are generated using directionally adaptive smoothness constraints *C*^*θ*^(*u*, *v*) according to the estimated edge orientation. To remove blurring artifacts caused by the interpolation process, the proposed restoration method performs 2D convolution using five 5 × 5 directionally adaptive TCLS filters according to the edge orientation *θ* as
22

where ∗ represents the 2D convolution operator, *ĝ*(*x*, *y*) is the interpolated image,  is the impulse response of the directionally TCLS filter of orientation *θ*, and  is the restored HR image.

## Experimental results

For evaluating the performance of the proposed digital zooming method, we used a set of standard images of size 512 × 512, and outdoor test images of size 1280 × 720 acquired by a mobile phone camera. The performance of the proposed method is evaluated with PSNR, SSIM and processing time in seconds on a personal computer with 3.4 GHz CPU and 8GB memory.

**Figure 6:**
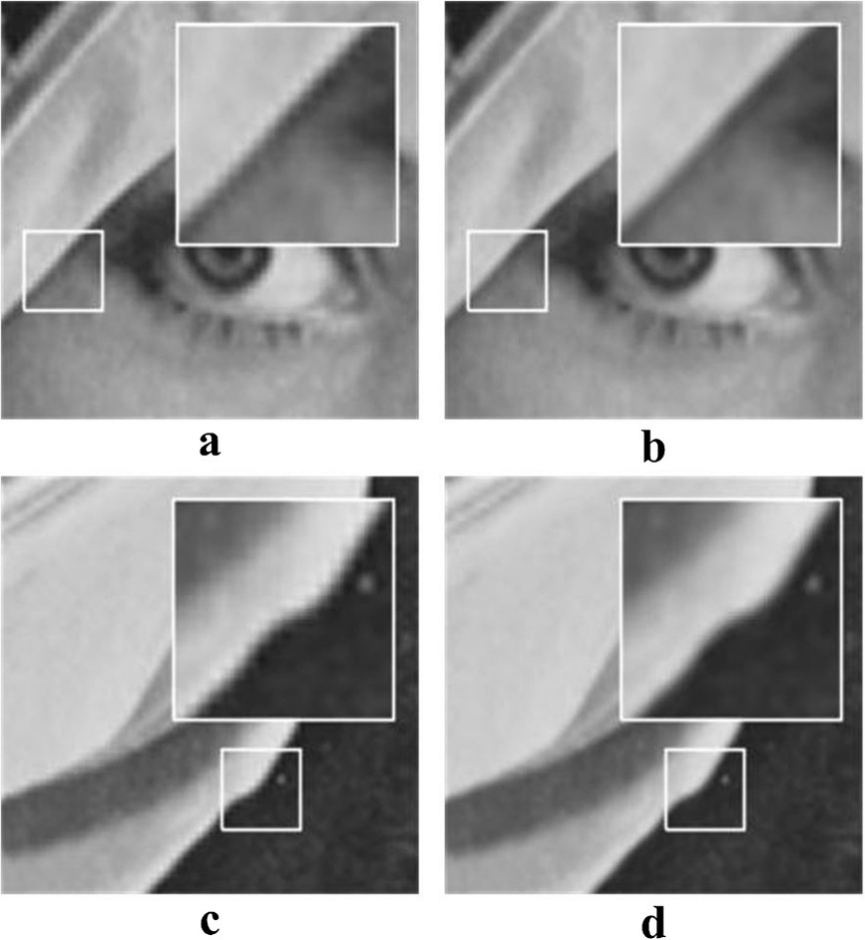
**Experimental results for the eight times magnification. a** and **c** cubic-spline interpolation with *a* = - 0.5. **b** and **d** the proposed method.

To evaluate the performance of the proposed digital zooming system by comparing with cubic-spline interpolation, the standard images and its eight times magnified version are used. The cubic-spline interpolation uses the weight function with *a* = - 0.5. The magnification results show that the proposed method can better remove jagging and blurring artifacts in the slanted edge region than the cubic-spline interpolation method as shown in Figure 6.

For additional experiments, to evaluate the performance and speed of the six interpolation algorithms such as cubic-spline interpolation, Li’s method (Li et al. 2001), Zhang’s method (Zhang et al. 2006), Giachetti’s method (Giachetti et al. 2011), Zhou’s method (Zhou et al. 2012), and the proposed method, standard images are generated by down-sampling 512 × 512 images by a factor of two in both horizontal and vertical directions.

Table 3 shows PSNR, SSIM and processing time for the two times magnification. These evaluation results show that the proposed method provides faster processing time than advanced interpolation method. Since the proposed method is based on the cubic-spline interpolation, it shows almost the same computational complexity as the original cubic-spline interpolation as shown in Table 3. However, the advanced interpolation algorithms use the iterative method to interpolate a new pixel for the magnification. Therefore, these methods needs higher computational complexity than the original cubic-spline interpolation and the proposed methods.

**Table 3:** **PSNR, SSIM, and CPU times of two interpolation methods using standard test images**

Image Type	Interpolation Type	PSNR	SSIM	Time (Sec)
Lena	Cubic-spline	33.7420	0.9693	0.281
	Li’s method	33.9460	0.9729	16.286
	Zhang’s method	33.8423	0.9723	12.979
	Giachetti’s method	34.0461	0.9701	97.249
	Zhou’s method	34.3944	0.9851	3.462
	Proposed method	34.1497	0.9728	0.608
Barbara	Cubic-spline	23.9285	0.8873	0.281
	Li’s method	22.1196	0.8665	17.006
	Zhang’s method	25.1406	0.8989	13.058
	Giachetti’s method	22.9126	0.8691	101.076
	Zhou’s method	23.3526	0.9851	3.452
	Proposed method	24.3669	0.8919	0.405
Boat	Cubic-spline	28.9056	0.9320	0.296
	Li’s method	29.3233	0.9393	17.160
	Zhang’s method	29.3349	0.9401	13.151
	Giachetti’s method	28.8491	0.9329	101.463
	Zhou’s method	29.5240	0.9835	3.439
	Proposed method	29.2777	0.9388	0.390
Crowd	Cubic-spline	21.6893	0.9370	0.281
	Li’s method	21.8357	0.9356	17.709
	Zhang’s method	22.2017	0.9409	12.963
	Giachetti’s method	21.7158	0.9367	99.841
	Zhou’s method	22.2346	0.7517	3.347
	Proposed method	22.0536	0.9414	0.460

For additional experiments, 1280 × 720 high-definition (HD) images are generated from 320 × 180 LR mobile camera images by four times magnification using the six advanced interpolation algorithms.

**Figure 7:**
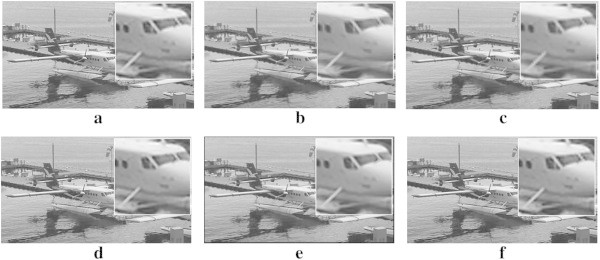
**Experimental results for the four times magnification from** 320 × 180 **LR mobile camera images. a** cubic-spline interpolation. **b** Li’s method. **c** Zhang’s method. **d** Giachetti’s method. **e** Zhou’s method. **f** the proposed method.

As shown in Figure 7b, strong interpolation artifacts are observed in Li’s method. Zhang’s method results in both jagging and blurring artifacts near edge regions as shown in Figure 7c. Giachetti’s method results in missing pixels near edge regions as shown in Figure 7d. Zhou’s method results in blurring artifacts near edge regions as shown in Figure 7e. The proposed method can successfully remove both jagging and blurring artifacts as shown in Figure 7f. Additional experimental results show that the proposed method provides higher quality magnification results without jagging and blurring artifacts than existing advanced interpolation algorithms.

## Conclusion

This paper presents novel directionally adaptive image interpolation and restoration algorithms for a fast digital zooming system in digital cameras. The proposed interpolation algorithm analyzes the edge orientation using computationally efficient steerable filters followed by the edge refinement process. The selective use of directionally weighted 1D interpolation and 2D adaptive cubic-spline interpolation can enhance the image quality without jagging artifacts in the slanted edge region. Blurring artifacts are also removed using directionally adaptive TCLS filters. Both proposed steerable filter-based interpolation and the TCLS-based restoration filters have an FIR structure for real-time processing in an ISP chain. Experimental results show that the proposed method can provide high-quality magnified images without jagging and blurring artifacts. Furthermore, the proposed method gives higher PSNR and SSIM values than existing state-of-the-art interpolation methods with reduced computation load.
